# Correlative STED and Atomic Force Microscopy on Live Astrocytes Reveals Plasticity of Cytoskeletal Structure and Membrane Physical Properties during Polarized Migration

**DOI:** 10.3389/fncel.2017.00104

**Published:** 2017-04-19

**Authors:** Nathan Curry, Grégory Ghézali, Gabriele S. Kaminski Schierle, Nathalie Rouach, Clemens F. Kaminski

**Affiliations:** ^1^Chemical Engineering and Biotechnology, University of CambridgeCambridge, UK; ^2^Center for Interdisciplinary Research in Biology, College de France, CNRS UMR 7241, INSERM U1050, Labex Memolife, PSL Research UniversityParis, France; ^3^Doctoral School No 158, Pierre and Marie Curie UniversityParis, France

**Keywords:** astrocytes, migration, protrusions, membrane physical properties, cytoskeleton, atomic force microscopy, STED, superresolution

## Abstract

The plasticity of the cytoskeleton architecture and membrane properties is important for the establishment of cell polarity, adhesion and migration. Here, we present a method which combines stimulated emission depletion (STED) super-resolution imaging and atomic force microscopy (AFM) to correlate cytoskeletal structural information with membrane physical properties in live astrocytes. Using STED compatible dyes for live cell imaging of the cytoskeleton, and simultaneously mapping the cell surface topology with AFM, we obtain unprecedented detail of highly organized networks of actin and microtubules in astrocytes. Combining mechanical data from AFM with optical imaging of actin and tubulin further reveals links between cytoskeleton organization and membrane properties. Using this methodology we illustrate that scratch-induced migration induces cytoskeleton remodeling. The latter is caused by a polarization of actin and microtubule elements within astroglial cell processes, which correlates strongly with changes in cell stiffness. The method opens new avenues for the dynamic probing of the membrane structural and functional plasticity of living brain cells. It is a powerful tool for providing new insights into mechanisms of cell structural remodeling during physiological or pathological processes, such as brain development or tumorigenesis.

## Introduction

Astrocytes are dynamic and motile signaling elements of the brain (Haber et al., 2006; Bernardinelli et al., 2014; Ghézali et al., 2016). They undergo intense morphological maturation during development, changing from sparsely branched cells to polarized motile and massively ramified cells (Bayraktar et al., 2014; Molofsky and Deneen, 2015). The dynamic remodeling of cytoskeleton architecture and membrane properties of glial cells is likely to play an important role in the establishment of cell polarity, adhesion and migration in various physiological and pathological conditions.

The cytoskeleton is composed of a meshwork of protein fibers, including actin microfilaments, microtubules and intermediate filaments (Fletcher and Mullins, 2010). Astrocytic morphological plasticity depends to a great extent on actin and tubulin (Goldman and Abramson, 1990; Etienne-Manneville, 2004, 2013; Haber et al., 2006). Both cytoskeletons drive changes in cell shape and motility by providing protrusive and contractile forces, as well as by supporting cellular trafficking. However the complex interplay between actin and tubulin structures in these processes remains elusive (Small et al., 1999; Etienne-Manneville, 2004; Carlier et al., 2015). The intermediate filaments, composed in particular of the glial fibrillary acidic protein (GFAP), also play an important role in the dynamic morphology of astrocytes in normal and pathological processes (Ridet et al., 1997). Nevertheless, in contrast to actin and tubulin, GFAP is excluded from fine peripheral astrocyte processes (Haseleu et al., 2013) and does not extend up to the leading edge of protrusions in migrating astrocytes (Sakamoto et al., 2013).

Thus the specific contribution of the various cytoskeleton elements to the functional plasticity of intrinsic cellular mechanical properties during dynamic processes such as migration still remains unclear (Etienne-Manneville, 2004). Several approaches have been used to characterize cytoskeletal organization in migrating cells, such as confocal microscopy or superresolution localization microscopy (Zhao et al., 2013; Zobel and Bogdan, 2013; van den Dries et al., 2013; Finkenstaedt-Quinn et al., 2016). Fluorescence imaging techniques have the advantage of allowing identification of defined subcellular elements. Alternatively, cellular mechanical properties such as stiffness or elasticity can be probed by atomic force microscopy (AFM) or optical tweezers-based techniques (Ritort, 2006; Haase and Pelling, 2015). A few studies have combined fluorescence imaging with AFM to simultaneously identify subcellular elements and probe cell mechanics (Haase and Pelling, 2015). However, the resolution limit of conventional approaches prevents identification of small filaments. Direct correlation of AFM images of mechanical properties with images of fine fluorescently labeled cytoskeletal structures therefore requires superresolution microscopy. AFM and superresolution microscopy has been previously combined on fixed cells (Chacko et al., 2013). However combining such techniques in living, intact cells remains challenging and has not been attempted yet. This is particularly relevant for investigating the contribution of fine cytoskeletal elements to the plasticity of cell mechanics occurring during polarized migration in live astrocytes. Investigating the dynamics of the various cytoskeletal elements and their role in cell shape and motility is challenging. There are currently very few organic dye markers for the proteins forming such structures that can be used for superresolution microscopy in live cells, as well as only a limited number of specific pharmacological tools available for their acute manipulation. Among the cytoskeletal elements, actin and tubulin are among the very few proteins that can be both visualized by superresolution stimulated emission depletion (STED) imaging using such live cell markers and be manipulated by pharmacological agents disrupting their assembly in microfilaments and microtubules.

We thus describe here a method to simultaneously characterize the organization of actin and tubulin cytoskeletal elements in astrocyte subcellular compartments and assess their involvement in cell topography and mechanical properties under normal conditions or during migration *in vitro*, applying correlative STED/AFM on live labeled astrocytes. We found that actin networks are highly organized in control cells. These networks are well reflected in cell topography and are key determinants of membrane stiffness. In contrast, tubulin elements do not exhibit such specific organized patterns and they do not contribute significantly to astrocyte stiffness. Remarkably, polarized migration induced the remodeling of both actin and tubulin cytoskeleton, which altered membrane stiffness specifically in fine astroglial protrusions. These data suggest that a redistribution of molecular complexes takes place in defined subcompartments that are involved in dynamic processes. The method we describe here can be used to provide directly correlated information at the molecular, structural and functional levels. This approach should help in future studies to provide further insights into the remodeling occurring in brain cells during physiological or pathological processes, such as development or tumorigenesis.

## Materials and Methods

### Animals

Experiments were carried out according to the guidelines of the European Community Council Directives of January 1st 2013 (2010/63/EU) and of the local animal welfare committee, and all efforts were made to minimize the number of animals used and their suffering.

### Primary Astrocyte Cultures and Scratch-Induced Migration Assay

Primary cortical astrocyte cultures were prepared as previously described (Koulakoff et al., 2008). Briefly, brains were removed from CD1 newborn pups (P1-P3) and the cortices were dissected in cold PBS-glucose (33 mM). Meninges were carefully withdrawn and cortices were mechanically dissociated. Astrocytes were seeded on poly-ornithine coated glass coverslips in DMEM containing 10% fetal calf serum, 10 U/ml penicillin, and 10 μg/ml streptomycin (GIBCO) and incubated at 37°C, 5% CO_2_. After 1 week, once cells have reached confluency, 1 μM of cytosine-arabinoside was added to the cell culture during 3 days to eliminate proliferating microglial cells. Medium was then changed every 3 days and cells were used after 2–3 weeks in culture. Confluent astrocytes were wounded by scraping monolayers with a 20 μl pipette tip (~300 μm in width) and imaged 6–8 h after the scratch (Etienne-Manneville, 2006).

### STED Microscopy

STED imaging was performed using a home built STED microscope system with pulsed excitation and depletion, and time-gated detection described previously (Mahou et al., 2015). In brief the system was developed on a commercial point scanning microscope (RESOLFT, Abberior Instruments) based around a microscope frame (IX83, Olympus), a set of galvanometer mirrors (Quad scanner, Abberior Instruments) and a detection unit consisting of two avalanche photodiodes (SPCM-AQRH, Excelitas Technologies). Images were acquired with a 100X/1.4 NA oil immersion objective lens (UPLSAPO 100X, Olympus). STED excitation and depletion pulses are generated from the same titanium-sapphire oscillator (Mai Tai HP, Spectraphysics) operating at 765 nm. Laser power is divided between two optical paths. In the excitation path a supercontinuum is generated by pumping a photonic crystal fiber (SCG800, NKT photonics). The excitation wavelength is selected from this supercontinuum using a bandpass filter. Depletion pulses are taken directly from the titanium sapphire (*λ* = 765 nm). Both excitation and depletion pulses are dispersed to durations of 50–70 ps and 100–300 ps respectively through propagation in polarization maintaining single mode fibers. The arrival times are synchronized using an optical delay line. The depletion beam is spatially shaped into a vortex beam using a spatial light modulator (SLM; X10468-02, Hamamatsu). The Fluorescence emission was descanned. A custom dichroic mirror (DM; Abberior Instruments) and bandpass filter (ET685-70) were used. The depletion pattern is generated by displaying a helical phase mask on the SLM. This is also used to correct for aberrations particularly defocus and spherical aberrations induced by cell culture medium. Acquisition was controlled using the Imspector Image Acquisition software (Andreas Schönle, Abberior Instruments GmbH, Göttingen, Germany). STED excitation power was 10–20 μW and depletion power was 100–150 mW (measured at the back aperture). The pixel size used throughout was 50 nm (*xy*) and 500–750 nm (*z*, depending on cell thickness) cell thickness was between 2–3 μm. Maximum intensity projections are presented throughout. Time gates of 1.719 ns after the excitation pulse were used.

Confocal images of GFP transfected cells were acquired after STED imaging on the same set up. Fluorescence excitation is from a 488 nm laser source (Cobolt 06-MLD-488 nm, Cobolt). A DM (zt 594 RDC, Chroma) and emission filter (Brightline HC 550/88, Semrock) are used in detection.

### Atomic Force Microscopy

AFM was performed on a commercial system (Bioscope RESOLVE, Bruker) and all measurements were performed using Nanoscope software (Bruker). PeakForce QNM-Live Cell probes (PFQNM-LC, Bruker AFM probes) were used throughout (Schillers et al., 2016). The deflection sensitivity of the probe was measured at the start of the experiment *in situ*, the spring constant of the probe was pre-calibrated and the no-touch calibration option in nanoscope software was used. Cells were imaged in the culture medium containing 10% HEPES. The sample stage was heated at 37°C. The AFM imaging mode was “Scanasyst in fluid” from the Nanoscope software—live cell and peak force set points are chosen between 200–600 pN for optimal force measurements. Typical values are 300 pN.

### Correlative STED/AFM

In this work a sample scanning AFM is used and fluorescence images are obtained from scanning the STED beam over the sample using galvanometric mirrors. The MIROview software (Nanoscope, Bruker) is used for correlative imaging. First the pixel sizes for optical imaging and AFM imaging are calibrated. The AFM translates the sample between three known *xy* positions and a fluorescent bead sample is imaged in each position. A common feature in each image is selected by the user. During imaging the AFM tip is manually centered on the optical field of view by translating the AFM stage. An optical image of the sample is then acquired. This image is imported into the MIROview software and AFM fields of view are selected on the optical image. Features which appear in both imaging modalities (e.g., borders of cells) are used as fiduciary markers and the optical image is translated, in software, such that both images are overlaid.

### Image Processing and Analysis

STED and confocal images are deconvolved using the Richardson-Lucy algorithm (deconvolution lab program in FIJI). The STED point spread function (PSF) is an average of PSFs measured using 20 nm beads. The confocal PSF is simulated using the package “PSF generator” available for FIJI.

Second order flattening is applied to AFM topography images to remove tilt and bow.

Stiffness values quoted are an estimate of the cone-sphere modulus taking into account the AFM tip size, shape and deflection sensitivity. Average values are measured for an 18 μm field of view (256 × 256). Where the field of view includes two cells then separate averages are taken for each cell. Values quoted are the average of all stiffness measurements in that cell. Fibritool analysis is used to measure the directionality of actin and tubulin filaments in images as an angle between −90° and 90°.

### Statistics

All data are expressed as mean ± SEM obtained from at least three independent experiments. *n* refers to the number of cells. Statistical significance for between-group comparisons was determined by unpaired or paired *t*-tests. Mann Whitney non parametric test has been used for discrete variables. Statistical analysis was performed in GraphPad InStat.

## Results

### Correlative STED-AFM Imaging of Live Astrocytes

**Figure 1:**
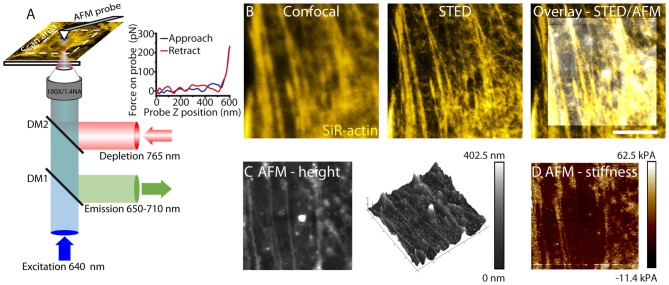
**Correlative stimulated emission depletion (STED)/atomic force microscopy (AFM) imaging. (A)** Schematic of STED/AFM imaging set-up. Fluorescence excitation pulses (640 nm) are combined with depletion pulses (765 nm) using a dichroic mirror (DM2, 730 nm short-pass). The depletion pulse is spatially shaped by an spatial light modulator (SLM) into an aberration corrected vortex beam. Fluorescent emission is separated using a dichroic mirror (DM1, custom). AFM images are acquired after STED imaging. AFM images are acquired pixelwise by translating the sample. The AFM cantilever is aligned such that STED and AFM have a common 80 × 80 μm scan area. For each pixel a force curve is measured by approaching the tip toward the sample and recording the tip-sample interaction force as a function of the cantilever z-position. An example force curve is shown in the inset. The Young’s modulus is estimated from the gradient. **(B)** STED microscopy (center) enhances resolution of actin filaments relative to confocal microscopy (left), revealing fine filamentous structure. The STED image is overlaid with an AFM height profile (right). **(C)** The sample height (left) as measured by AFM. The color scale represents the range in sample height between 0 nm and 402.5 nm. Cytoskeletal components are detected through the cell membrane. The height image is also represented as a 3D map. **(D)** Young’s modulus (stiffness) is shown. The color scale ranges from −11.4 kPa to 62.5 kPa and represents values measured from the force curve.

The STED microscope was developed for live cell imaging (Figure 1A). In particular, a pulsed laser at 765 nm was chosen for depletion, resulting in reduced phototoxicity as cells have low autofluorescence at this wavelength. To compensate for spherical aberrations induced by the change in refractive index between immersion oil and cell culture medium, a SLM was used to precompensate for wavefront aberrations. This system has been previously reported and achieves resolutions of up to 50 nm (Mahou et al., 2015; Fusco et al., 2016). Live cells were labeled with SiR-actin or SiR-tubulin (Lukinavičius et al., 2014). These organic dye labels have been previously reported as STED dyes and feature low cytotoxicity and high photostability for imaging intact cells. STED images allow the resolution of individual actin or tubulin filaments, compared to confocal microscopy (Figure 1B).

For live cell AFM imaging (Figures 1C,D), probes were chosen for imaging cells with varying heights. PeakForce QNM-Live cell probes (PFQNM-LC, Bruker AFM probes) were used due to their 17 μm long tips which allows imaging of structures with varying heights without the cantilever coming into contact with the sample (Schillers et al., 2016).

AFM measurements use the PeakForce QNM mode. This mode minimizes the forces applied by the cantilever to the sample and acquires a force curve for each pixel (Figure 1A). A map of both height and stiffness (as the Young’s modulus) can be simultaneously measured from this imaging mode (Figures 1C,D). The AFM probe sensitive to structures below the cell membrane reveals a polarized structure in the height image. Stiffness measurements are measured simultaneously with height images and therefore directly correlate.

For correlative imaging the cantilever is mounted and the AFM scan head is translated such that it is centered on the STED field of view. STED images of the sample are acquired prior to AFM images. In our experimental set-up, height images can be correlated with optical images of the cytoskeleton for an 80 × 80 μm field of view. This allows a direct correlation of cytoskeletal structure with both topography and membrane stiffness for living astrocytes.

### Actin Cytoskeleton Correlates with Astrocyte Topography and Membrane Physical Properties

**Figure 2:**
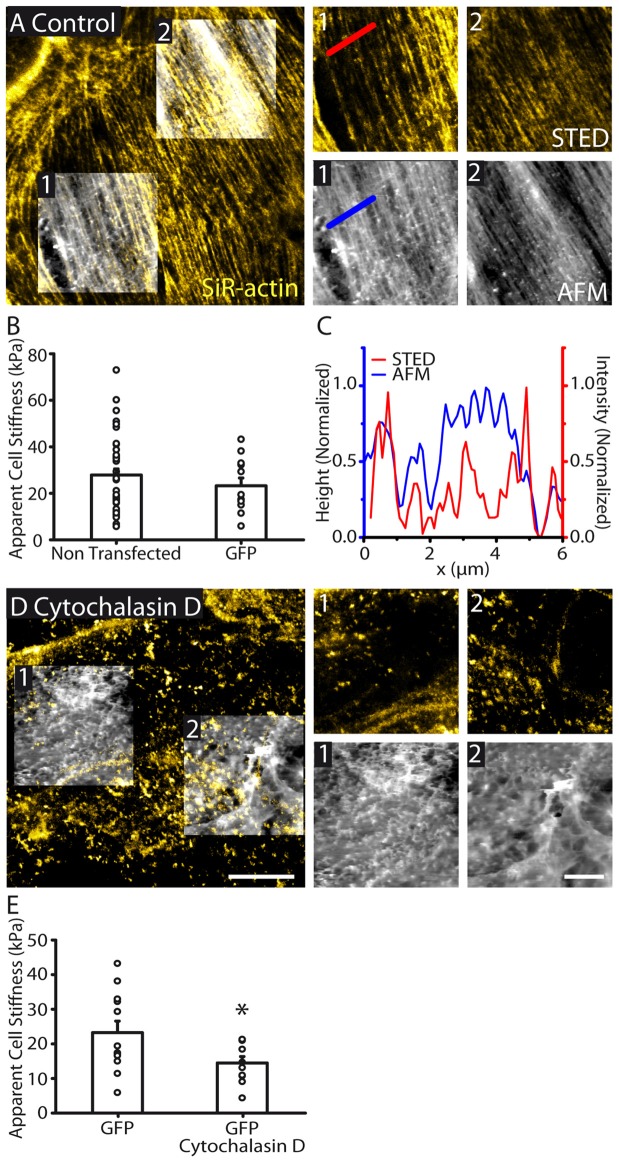
**Actin cytoskeletal structure is reflected in cell topography and cellular stiffness. (A)** STED image of the actin cytoskeleton overlaid with AFM topography (height) images (large image). AFM images were taken in regions 1 and 2. Zoomed AFM and the corresponding STED images are shown. STED/AFM reveals an agreement between the fluorescently labeled actin filaments and AFM topography. Both imaging modes show similar polarity. AFM color scale represents a range of heights of 455 nm and 464 nm in regions 1 and 2 respectively. **(B)** Bar graph representing the mean cell stiffness of control (non transfected, *n* = 38) and GFP transfected astrocytes (*n* = 12), showing no significant effect of GFP transfection on stiffness (*p* > 0.05, Mann Whitney test). **(C)** Line profile through the indicated region showing height (AFM) and signal (STED) showing good agreements between the locations of peaks in both profiles. **(D)** STED and AFM height images (height range: region 1; 1394 nm, region 2; 1320 nm) of an astrocyte with cytoskeleton depolymerized by cytochalasin D (30 μM, 1 h). STED images reveal actin depolymerization, while AFM images show reduced organization in cell topography. **(E)** Depolymerizing actin reduces cytoskeletal stiffness (GFP, *n* = 12; GFP + cytochalasin D, *n* = 10, *p* < 0.05, unpaired *t* test). Asterisk indicates statistical significance (*p < 0.05). Scale bars are 10 μm (large images) and 5 μm (zoomed images).

We first simultaneously assessed astrocyte topography and membrane physical properties using AFM. AFM height images revealed highly polarized structural elements within *in vitro* astrocytes (Figure 2A). In addition, probing astrocyte surface with AFM generated maps of stiffness throughout individual astrocytes, and revealed a mean cell stiffness of 27.9 ± 2.6 kPa (*n* = 38, Figure 2B). Notably, a wide range of cell stiffnesses was observed, likely reflecting astrocyte heterogeneity. Cell labeling with GFP transfection enabled clear identification of individual astrocytes, and did not alter apparent cell stiffness (23.2 ± 3.3 kPa, *n* = 12, *p* > 0.05, Mann Whitney test, Figure 2B).

To identify the nature of the topographic structures (Figure 2A), we simultaneously performed STED imaging of actin cytoskeleton using SiR-actin dye in live astrocytes. We found a similarly polarized distribution of actin fibers within confluent astrocytes (Figure 2A). In addition, actin was also highly organized in array of fibers throughout individual astrocytes. Actin fibers revealed by STED imaging strongly correlated with the astrocyte topology assessed with AFM (Figures 2A,C). In addition, actin fibers and the structural elements from the AFM display similar orientation, as assessed by Fibritool analysis (region 1: AFM: 67.2°, STED: 68.8°; region 2: AFM: 56.6°, STED: 57.4°, Figure 2A). To test the contribution of actin cytoskeleton to astrocyte membrane physical properties, we disrupted actin networks in astrocytes using cytochalasin D (30 μM, 1 h; Figure 2D). GFP labeling enabled delineating individual astrocytes and co-alignment of fluorescence and AFM images. We found that actin disruption decreases astrocyte stiffness (GFP: 23.2 ± 3.3 kPa, *n* = 12; GFP + cytochalasin D: 14.5 ± 1.9 kPa, *n* = 10, *p* < 0.05, unpaired *t* test, Figure 2E). Altogether these data show that actin cytoskeleton contributes to astrocyte topography and membrane physical properties.

### Tubulin Cytoskeleton Pattern Does Not Reflect Astrocyte Surface Profile and Stiffness

**Figure 3:**
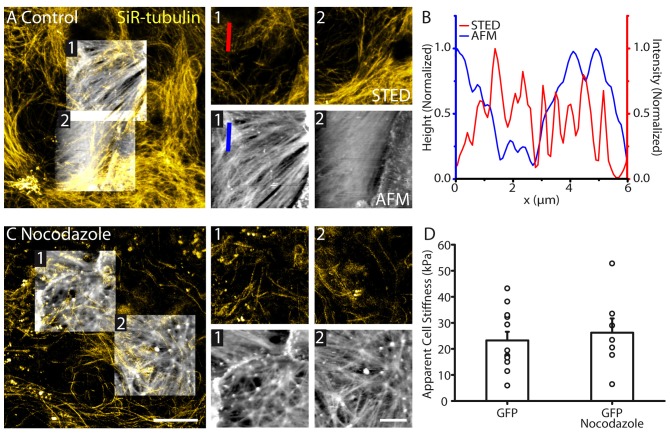
**Astrocyte surface topography and stiffness is distinct from tubulin organization. (A)** Correlative STED/AFM images of tubulin cytoskeleton and astrocyte topography. AFM images (height range: region 1; 279 nm, region 2; 389 nm) reveal a structure with different polarity to the corresponding STED images of tubulin region 1: AFM: 32.2°, STED: 51.3°; region 2: AFM: 29.0°, STED: 40.0°. **(B)** Line profile through the indicated region of the AFM (height) and STED (intensity) showing little agreement between peak locations and structures. **(C)** STED and AFM images of astrocytes (AFM height range: region 1; 680 nm, region 2; 388 nm) where tubulin has been depolymerized using nocodazole (16 μM, 1 h). Fibrilar structures remain present in the topography images. **(D)** No change in average stiffness was detected between control astrocytes (*n* = 12) and astrocytes treated with nocodazole (*n* = 7, *p* > 0.05, Mann Whitney test). Scale bars are 10 μm (large images) and 5 μm (zoomed images).

To investigate the involvement of tubulin on astrocyte topography and physical membrane properties, we performed STED-AFM recordings, where tubulin was labeled using SiR-tubulin dye. The tubulin cytoskeleton displayed a different structure compared to actin fibers, with no clear polarized orientation within astrocytes (Figure 3A). Tubulin filaments imaged with STED did not correlate with astrocyte topology revealed by AFM (Figures 3A,B). In addition, tubulin cytoskeleton showed a different orientation compared to the structural elements from the AFM (region 1: AFM: 32.2°, STED: 51.3°; region 2: AFM: 29.0°, STED: 40.0°, Figure 3A). To test the involvement of tubulin elements in astrocyte stiffness, we depolymerized tubulin structures in astrocytes using nocodazole (16 μM, 1 h; Figure 3C). GFP labeling enabled identification of individual astrocytes despite loss of tubulin structure. In contrast to actin, tubulin elements had no effect on astrocyte stiffness (GFP: 23.2 ± 3.3 kPa, *n* = 12; GFP + nocodazole: 26.2 ± 5.5 kPa, *n* = 7, *p* > 0.05, Mann Whitney test, Figure 3D).

### Polarized Migration Alters Cytoskeleton Distribution and Subcellular Stiffness in Astrocyte Protrusions

**Figure 4:**
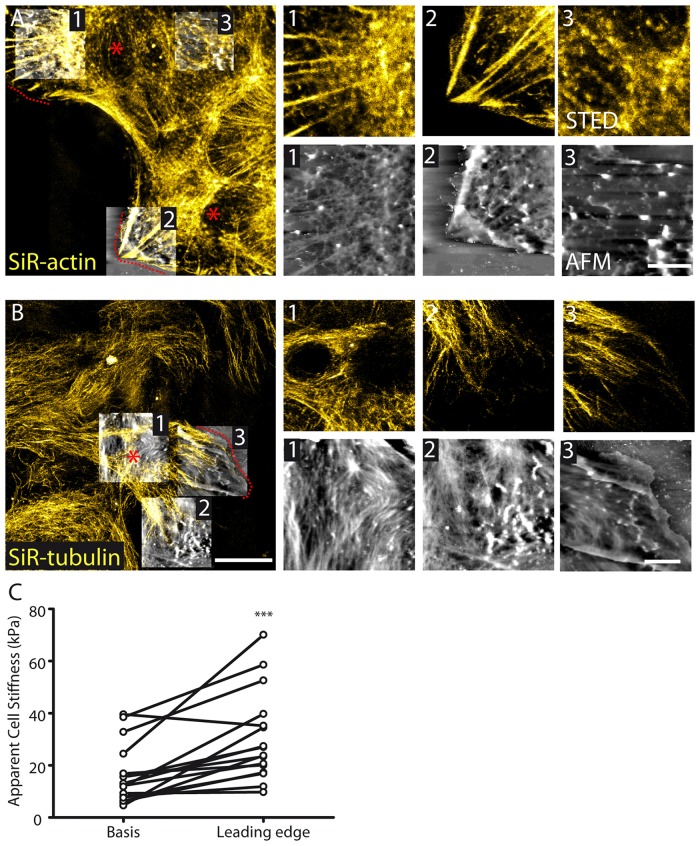
**Cytoskeletal and membrane stiffness reorganization in migrating astrocytes. (A)** STED images reveal polarized stress fibers in the direction of migration with corresponding fibers appearing in AFM images 1 and 2. At the basis of the protruding cell (e.g., AFM image 3) actin organization appears to be reduced in both STED and AFM images (AFM height range: region 1; 564 nm, region 2; 814 nm, region 3; 942 nm). **(B)** Tubulin appears polarized in the direction of migration in astrocytes (AFM height range: region 1; 297 nm, region 2; 322 nm, region 3; 396 nm). The dash lines and asterisks (red) indicate the leading edges and bases, respectively, of the protrusions. **(C)** Cellular stiffness is increased at the leading edge of protruding cells relative to the basis center of the protrusion (*n* = 16, *p* < 0.001, paired *t* test). Asterisks indicate statistical significance (***p < 0.001).

Cytoskeleton remodeling occurs in migrating astrocytes. To induce astrocyte polarized migration, we used the well-established scratch assay in cultured astrocytes (Etienne-Manneville, 2006). Such assay generates astrocytes protrusions migrating in a direction perpendicular to the scratch through a well-defined integrin signaling activating small G-proteins (Etienne-Manneville and Hall, 2001). We found that during scratch-induced migration, actin fibers concentrate in a polarized fashion at the leading edge of astrocyte migrating protrusions (Figure 4A). Noteworthy, this was associated with a reduced organization of actin at the basis of the protrusions (Figure 4A). On the contrary, tubulin showed increased organization characterized by polarization of filaments in the direction of the migration both at the basis and at the leading edge of the protrusion (Figure 4B). To decipher whether cytoskeleton remodeling induced by polarized migration alters at the subcellular level membrane properties of astrocyte protrusions, we performed membrane stiffness measurements using AFM. We found that stiffness was increased at the leading edge of astrocyte protrusion (30.5 ± 4.3, *n* = 16) compared to its basis (16.3 ± 2.9, *n* = 16, *p* < 0.001, paired *t* test, Figure 4C).

## Discussion

We present a technique that combines superresolution microscopy and AFM on live astrocytes to correlate subcellular cytoskeletal organization with cell morphology and membrane stiffness. The method is well-adapted to studying the distribution of various subcellular components and to correlate this information with functional mechanical properties of specific microdomains. The addition of optical superresolution goes beyond previous work, which focused purely on AFM as a readout (Haase and Pelling, 2015; Finkenstaedt-Quinn et al., 2016; Lemière et al., 2016; Luo et al., 2016) and permits one to correlate the spatial distribution of labeled cytoskeletal elements, identified with unprecedented resolution even in dense meshworks, to cell topography. In previous studies standard confocal microscopy was reported in combination with AFM recordings to investigate cell topography or membrane physical properties on fixed cells, but was not used to infer a direct connection between cytoskeleton elements, cell topography and membrane properties in living cells. The technique that we here describe is used to image live cells in superresolution ensuring that AFM readouts of functional properties are from intact cells.

Using this technique, we found that actin cytoskeleton is highly organized in astrocytes especially in those featuring polarization. We found parallel actin filament bundles in fine protrusions at the leading edge of migrating living cells. Since the dynamics of actin is a key determinant of cell motility (Pollard and Cooper, 2009; Carlier et al., 2015; Sens and Plastino, 2015), such bundles are likely there to promote adhesion, as well as contraction and growth of the protrusions mediating migration (Bornschlögl, 2013). Remarkably, we see that the actin cytoskeleton is also well reflected in the cell topography, which also displays highly polarized parallel features (Yamane et al., 2000). Polarized actin bundles are indeed clearly visible on AFM images, and represent the majority of the structural elements revealed by AFM. Actin also contributes significantly to measured cell stiffness in basal conditions, as the selective pharmacological disruption of actin networks in cultured astrocytes induced a decrease in basal membrane stiffness. This is in line with other studies performed in different cell types describing a close relationship between cellular stiffness and actin architecture (Roduit et al., 2009; Mihai et al., 2012; Pogoda et al., 2012; Louise et al., 2014; Ramos et al., 2014). Remarkably, in maturing astrocytes the increased density of cytoskeletal proteins such as actin, tubulin or GFAP also positively correlates with a rise in their membrane stiffness during development (Lee et al., 2014). This raises the intriguing possibility that cytoskeletal elements other than actin and tubulin, such as intermediate filaments or actin-binding proteins like ezrin, known to be enriched in astroglial protrusions (Haseleu et al., 2013), may also contribute to membrane stiffness and contrast in the AFM images.

Interestingly, combining STED with AFM also revealed that tubulin patterns differ from those of actin in astrocytes, and tubulin displays a non-polarized structure in control condition. In addition, we also found that the astroglial tubulin cytoskeleton did not significantly contribute to cell stiffness under basal conditions. Although microtubules are stiffer than actin, and are recognized to play a major role in cell migration via modulation of cell mechanics and intracellular trafficking (Etienne-Manneville, 2004, 2013; Luo et al., 2016), their contribution to cell stiffness has been less studied than that of actin. In contrast to our finding in control astrocytes, microtubules have been reported to play an important role in neuronal stiffness at the axonal level (Ouyang et al., 2013). Together, these data suggest that microtubule contribution to cell mechanics may be cell or compartment specific, and be particularly prominent during dynamic processes such as cell migration.

In fact, we found that migration induced remodeling not only of actin, but also of the tubulin cytoskeleton, in astrocytes. These changes altered astroglial stiffness at the subcellular level. Stiffness was particularly increased at the leading edge of astroglial polarized protrusions, compared to the protrusion basis, suggesting a significant contribution of actin. However, determining the specific role of actin vs. tubulin cytoskeleton in cell stiffness changes occurring subcellularly during migration is challenging, as both are remodeled and likely participate in the rearrangement of the other cytoskeleton (Etienne-Manneville, 2004). Coordinated interplay of actin and tubulin cytoskeleton in specific microdomains is indeed thought to be the key to changes in cell mechanics, enabling cell motility during polarized migration (Etienne-Manneville, 2004, 2013; Pollard and Cooper, 2009; Fletcher and Mullins, 2010; Haase and Pelling, 2015; Sens and Plastino, 2015). Thus, the simplified view that during migration actin bundles and stress fibers in the cell body and leading edge provide the driving force, whereas microtubules allow cellular trafficking via their polarized network throughout the cell has evolved: if filopodia and lamellopodia are unquestionably enriched in actin and hardly harbor any tubulin, the extension of microtubules towards the leading edge can still contribute to cell protrusion; either directly, through generation of a force at their plus end, or indirectly by controlling actin regulators promoting actin rearrangements. In addition, the actin and tubulin crosstalk during migration also involves dynamic regulation of focal adhesions, on which actin stress fibers are attached, and that controls cell contractility. Thus the current view is that both cytoskeletal elements are coordinately modulated by signaling molecules and directly regulate each other’s dynamics, so that their functions in cell shape, polarity and migration are actually overlapping (Small et al., 1999; Etienne-Manneville, 2004; Carlier et al., 2015).

In all, combining STED-AFM imaging is a powerful and promising approach in functional cell biology, permitting the identification of any labeled cellular structure using appropriate dye or labeling methods, such as SNAP-tag labeling (Hussain et al., 2013), and the relation to changes in cell topography and functional mechanical properties with subcellular resolution. The approach should be particularly interesting for efforts to decipher the molecular and cellular processes underlying the plasticity of cell mechanics that occur during cell migration, adhesion and division in physiological and pathological conditions.

## Author Contributions

NC, NR and GG performed the experiments. NC and NR the analyzed data and wrote the manuscript. NC, NR and CFK contributed to the design of the experiments. GSKS provided technical support.

## Conflict of Interest Statement

The authors declare that the research was conducted in the absence of any commercial or financial relationships that could be construed as a potential conflict of interest.
